# *In utero* ethanol exposure induces mitochondrial DNA damage and inhibits mtDNA repair in developing brain

**DOI:** 10.3389/fnins.2023.1214958

**Published:** 2023-08-09

**Authors:** Nune Darbinian, Armine Darbinyan, Nana Merabova, Myrna Kassem, Gabriel Tatevosian, Shohreh Amini, Laura Goetzl, Michael E. Selzer

**Affiliations:** ^1^Center for Neural Repair and Rehabilitation (Shriners Hospitals Pediatric Research Center), Lewis Katz School of Medicine, Temple University, Philadelphia, PA, United States; ^2^Department of Pathology, Yale University School of Medicine, New Haven, CT, United States; ^3^Medical College of Wisconsin-Prevea Health, Green Bay, WI, United States; ^4^Department of Biology, College of Science and Technology, Temple University, Philadelphia, PA, United States; ^5^Department of Obstetrics and Gynecology, University of Texas, Houston, TX, United States; ^6^Department of Neurology, Lewis Katz School of Medicine at Temple University, Philadelphia, PA, United States

**Keywords:** mitochondria, brain development, FASD, exosomes, mtDNA damage, IGF-1, mtDNA repair, 8-oxoguanine DNA glycosylase-1 (OGG1)

## Abstract

**Introduction:**

Mitochondrial dysfunction is postulated to be a central event in fetal alcohol spectrum disorders (FASD). People with the most severe form of FASD, fetal alcohol syndrome (FAS) are estimated to live only 34 years (95% confidence interval, 31 to 37 years), and adults who were born with any form of FASD often develop early aging. Mitochondrial dysfunction and mitochondrial DNA (mtDNA) damage, hallmarks of aging, are postulated central events in FASD. Ethanol (EtOH) can cause mtDNA damage, consequent increased oxidative stress, and changes in the mtDNA repair protein 8-oxoguanine DNA glycosylase-1 (OGG1). Studies of molecular mechanisms are limited by the absence of suitable human models and non-invasive tools.

**Methods:**

We compared human and rat EtOH-exposed fetal brain tissues and neuronal cultures, and fetal brain-derived exosomes (FB-Es) from maternal blood. Rat FASD was induced by administering a 6.7% alcohol liquid diet to pregnant dams. Human fetal (11–21 weeks) brain tissue was collected and characterized by maternal self-reported EtOH use. mtDNA was amplified by qPCR. OGG1 and Insulin-like growth factor 1 (IGF-1) mRNAs were assayed by qRT-PCR. Exosomal OGG1 was measured by ddPCR.

**Results:**

Maternal EtOH exposure increased mtDNA damage in fetal brain tissue and FB-Es. The damaged mtDNA in FB-Es correlated highly with small eye diameter, an anatomical hallmark of FASD. OGG1-mediated mtDNA repair was inhibited in EtOH-exposed fetal brain tissues. IGF-1 rescued neurons from EtOH-mediated mtDNA damage and OGG1 inhibition.

**Conclusion:**

The correlation between mtDNA damage and small eye size suggests that the amount of damaged mtDNA in FB-E may serve as a marker to predict which at risk fetuses will be born with FASD. Moreover, IGF-1 might reduce EtOH-caused mtDNA damage and neuronal apoptosis.

## 1. Introduction

Fetal ethanol (EtOH) exposure during pregnancy is the leading cause of preventable cognitive impairment. Alterations of grey and white matter integrity are consistent findings in fetal alcohol spectrum disorders (FASD), and their most severe form, fetal alcohol syndrome (FAS), although the molecular mechanisms involved in these abnormalities are still not well understood (May et al., 2009, 2014, 2018; Valenzuela et al., 2011; Hoyme et al., 2016). Unfortunately, many women use alcohol before they know they are pregnant Not all exposed fetuses develop FASD, but there is no way to predict which children will be born with FASD because imaging methods currently lack sufficient resolution, particularly early in pregnancy, when the pathogenesis most likely begins, and there are no established *in utero* molecular markers for FASD, even for FAS. Recently, it was demonstrated that EtOH exposure downregulated the expression of neuronal markers and markers of mature oligodendrocytes (OLs) in fetal brain in the mid-second trimester (Darbinian et al., 2021a, 2023). People with FAS may experience early aging, like people with some genetic disorders, and thus have reduced life spans (Moore and Riley, 2015). Mitochondrial dysfunction and mitochondrial DNA (mtDNA) damage, the hallmarks of aging, are postulated to be a central event in FASD (Hoek et al., 2002; Bukiya, 2019). If mtDNA damage is not repaired, oxidative stress and cell damage are increased, and mitochondrial function is impaired. Quantitative or qualitative alterations in mtDNA repair proteins, particularly 8-oxoguanine DNA glycosylase-1 (OGG1), may inhibit mtDNA repair. Biomarkers that can detect FASD early in pregnancy might suggest strategies that can improve the quality of life and life expectancy of affected people as they age.

### 1.1. Mitochondrial injury

Mitochondria are sources of increased levels of reactive oxygen species (ROS) in age-related neurodegenerative disorders (Kaarniranta et al., 2019; Gyllenhammer et al., 2022). ROS increase is correlated with mtDNA damage (Zhao and Sumberaz, 2020). Further, Human Immunodeficiency Virus 1 (HIV-1) Tat inhibits neuronal cell survival through a mitochondrial pathway, and impaired mitochondrial oxidative phosphorylation is an important feature of HIV-1 pathogenesis (Côté et al., 2002; Casula et al., 2005; De Simone et al., 2016; Darbinian et al., 2020). The mitochondrial genome encodes only 37 genes, whereas most mitochondrial proteins are regulated by nuclear DNA (nDNA) (Abdullaev et al., 2020). Old mitochondria are replaced by autophagy (mitophagy) (Thangaraj et al., 2018). The mitochondrial genome is 16.5 kb. Each cell has several thousand copies of mitochondrial DNA. In age-related neurodegenerative diseases, nDNA is less susceptibile to DNA-damaging agents tthan is mtDNA (Cline, 2012). After oxidative stress in human cells, damage to nDNA also is less extensive than to mtDNA (Zhao and Sumberaz, 2020). Damage of mtDNA is reversed by the base excision repair (BER) pathway. There is a correlation between ROS-induced mtDNA damage and reductions in activity of BER, including OGG1 (Kaarniranta et al., 2019).

### 1.2. Mitochondrial DNA

mtDNA impacts the central nervous system (CNS) and peripheral nervous system (PNS) (Carelli and Chan, 2014), and is involved in neurodegenerative disorders (Yang et al., 2008). Mitochondrial DNA copy numbers were reduced in pyramidal neurons, and mitochondrial biogenesis signaling was disrupted in hippocampuses of patients with Alzheimer’s disease (Rice et al., 2014). Mitochondrial changes also were detected in the aging human placenta (Bartho et al., 2020). HIV-1 and HIV-1-Tat can induce mtDNA damage in human neurons (Darbinian et al., 2020). Placental mtDNA content during development influences childhood intelligence (Bijnens et al., 2019). Prenatal exposures to cigarette smoke can alter nuclear and mitochondrial DNA in newborns (Pirini et al., 2015), and can increase oxidative stress and mitochondrial damage in non-human primates (Westbrook et al., 2010). In human cells, oxidative stress leads to more extensive and prolonged damage to mtDNA compared to nDNA (Yakes and Van Houten, 1997). Developmental changes in mtDNA also were found in human cord blood leukocytes during gestation (Pejznochová et al., 2008).

### 1.3. mtDNA damage and BER by OGG1

BER repairs oxidatively induced DNA base lesions in mitochondria, and consists of long and short patch pathways, involving multiple enzymes (Prakash and Doublié, 2015; Dizdaroglu et al., 2017). For example, OGG1 can protect against methamphetamine-enhanced fetal brain oxidative DNA damage (Wong et al., 2008). The first steps in the repair of 8-oxoG through the BER pathway are catalyzed by OGG1. OGG1 was expressed and activated in adult rodent brain tissues (Verjat et al., 2000). The oxidative DNA damage levels in the brain depend on the ability of OGG1 to remove 8-oxoG in mouse brain (Cardozo-Pelaez et al., 2000). BER of 8-hydroxyguanine protects DNA from endogenous oxidative stress (Boiteux and Radicella, 1999). Two major forms of human OGG1 are encoded by alternatively spliced OGG1 mRNAs (Nishioka et al., 1999). ROS can induce DNA damage, the product of OGG1 gene activation involved in this process is 8-hydroxyguanine (8-OH-G). OGG1 inactivation can cause spontaneous mutation, such as an increase in GC to TA transversions (Boiteux and Radicella, 2000). Another enzyme that regulates oxidative stress and aging is a longevity determing catalase (Cutler, 2005).

### 1.4. Exosomes

The brain cells of fetuses release small vesicles, exosomes, which carry cell components, and find their way into the mother’s blood, from which they can be isolated without disturbing the fetus. Circulating exosomes are strongly implicated in FASD (Goetzl et al., 2016, 2019a; Darbinian et al., 2023). Studies in rats and human tissues showed increased apoptosis, cytokine dysregulation and maturational defects in neurons and OLs. These molecular abnormalities are detectable in cell type-specific (i.e., OLs and neurons) FB-E isolated from maternal blood. The contents of these cell type-specific exosomes can serve as biomarkers for neuronal and OL damage in FASD. Not only mRNA (Valadi et al., 2007; Skog et al., 2008; Batagov and Kurochkin, 2013), but even double-stranded DNA can be found in exosomes (Thakur et al., 2014; Witwer et al., 2017), and exosomes secreted by OLs (Fruhbeis et al., 2012) contain major myelin and stress-protective proteins (Kramer-Albers et al., 2007), lipids (Skotland et al., 2017) and miRNAs (Ebrahimkhani et al., 2017). Recently mtDNA was found in exosomes (Vaidya et al., 2022), which also carry genomic and cytoplasmic DNA. Cells exposed to pathological conditions may secrete exosomes containing abnormal mtDNA (Vaidya et al., 2022), and this might also apply to damage from EtOH exposure. Considering the previous data (Darbinian et al., 2021a, 2023; Darbinian and Selzer, 2022), it would be possible that increased release of soluble factors may be involved in the dysregulation of OL and neuronal growth and survival. Changes in differentiation and chemokine secretion by OLs are associated with activation of apoptotic signaling in differentiated rat OL O2A cells and neurons (Darbinyan et al., 2013a,b; Darbinian et al., 2021a).

### 1.5. Fetal brain-derived exosomes (FB-Es) to study mitochondrial repair genes

Neurons and OLs are damaged in FASD, and either fail to develop, or undergo excessive apoptosis. Fetal brain tissue examination is not possible in ongoing human pregnancies, and non-invasive use of the fetal brain has been limited to expensive and technically challenging *in utero* imaging studies. Maternal plasma miRNAs have been used to predict infant outcomes and may be helpful to diagnose FASD subpopulations (Balaraman et al., 2014, 2016; Tseng et al., 2019). Previously, we determined the effect of prenatal EtOH exposure on human fetal neuronal and OL apoptosis (Amini et al., 2009; Darbinyan et al., 2013a; Darbinian et al., 2021a,b). Here we extend these studies to the effects of EtOH on mtDNA in FB-Es.

## 2. Methods

### 2.1. Clinical samples

We established a biobank that included 155 women who consumed EtOH, but no other drugs, during pregnancy, and had elective termination of their pregnancy due to economic or family conditions, and not because of adverse events, acute or chronic disease, or concerns about the health of the fetus. These were designated “clean EtOH-exposed” cases. We also incorporated 75 controls. EtOH-exposed and control cases were numbered in order of acquisition. For the present study, we selected the first 20 subjects and compared them to the first 20 carefully matched unexposed controls. Cases and controls were matched for sex and gestational age (GA) between 11- and 21-weeks GA (Table 1) under a protocol approved by our Institutional Review Board (IRB). All assays were performed in triplicates. Data from both sexes were combined. Details of the clinical samples, fetal brain tissues from elective termination of pregnancy, and matching maternal blood samples used in this study were reported earlier (Goetzl et al., 2016, 2019a; Darbinian et al., 2021a, 2023).

**Table 1:** Human subjects: Clinical characteristics.

	EtOH consumers (*n* = 20)	Controls (EtOH Non-consumers; *n* = 20)
Maternal Age (years ±SD)	28.0 ± 2.7	24.15 ± 2.3
Gestational Age (weeks ±SD)	15.22 ± 1.6	14.92 ± 1.58
Gestational Age range (weeks)	11.5 to 21	11.1 to 21
Ethnicity: Hispanic (%)	15	15
Race: White (%)	50	50
Race: Black (%)	50	50
Fetal Sex, Male (%)	50	50
Fetal Sex, Female (%)	50	50

#### 2.1.1. Subject recruitment

Women reporting alcohol use (or no alcohol use) since conception were grouped in GA windows: 11–21 weeks (1st - 2nd Trimester) (Darbinian et al., 2023). GA was determined by a dating ultrasound performed immediately prior to recruitment (Spong et al., 2011). Blood samples of 20 “clean EtOH-consuming” subjects and 20 matched controls were collected. For EtOH-consuming mothers, the total cumulative alcohol dose in 1st trimester pregnancy terminations ranged from 57–168 drinks (or 12–30 drinks/month), and for 2nd trimester terminations was from 54.4 to 1827.5 drinks (or 6–320 drinks/month). A drink was estimated as the equivalent of one shot [1.5 oz. of brandy or 5 oz. of wine (Darbinian et al., 2023)]. Due to the limited nature of this study, and for the need to match EtOH-exposed fetuses with appropriate controls, it was not possible to carry out the study in a completely blinded way. However, moment by moment, the samples were identified only by their accession number, and most of the tissue handling and testing was automated so as to analyze all the EtOH-exposed and control samples simultaneously on the same devices.

#### 2.1.2. Eligibility criteria

The brain and blood samples were obtained according to NIH Guidelines through a trained Study Coordinator. Samples were collected regardless of ethnic background, sex, and race.

#### 2.1.3. Treatment plan

Each patient signed a separate consent form for research on blood and tissue samples. Collected blood was processed for collection of plasma. No invasive procedures were performed on the mother, other than those used in her routine medical care. Fetal tissues were processed for RNA or protein isolation.

#### 2.1.4. Risk and benefits

There are very small risks of loss of privacy as with any research study where protected health information is viewed. The samples were depersonalized before they were sent to the lab for analysis. There was little anticipated risk from obtaining approximately 2–3 cc of blood, but a well-trained Study Coordinator.

There was no direct benefit to the research subjects from participation, but there is significant potential benefit for the future FASD subjects.

#### 2.1.5. Informed consent

Consent forms and the de-identified log sheets and IRB protocol were sent by the Study Coordinator to Principal Investigator with each blood and tissue sample. Except for an assigned accession number, no identification was kept on the blood and tissue samples (Darbinian et al., 2023).

#### 2.1.6. Assessment of alcohol exposure in pregnancy

Maternal EtOH exposure was determined with a face-to-face questionnaire (Goetzl et al., 2016, 2019a; Darbinian et al., 2021a, 2023; Darbinian and Selzer, 2022). EtOH exposure was assessed using a detailed questionnaire adapted from the NICHD PASS study (Dukes et al., 2014). Women admitting to EtOH use were classified as EtOH exposed (see all details in Darbinian et al., 2023).

#### 2.1.7. Tissue collection

Fetal brain tissue from subjects undergoing elective termination of pregnancy was collected according to an IRB-approved protocol (Goetzl et al., 2016; Darbinian et al., 2021a, 2023).

### 2.2. Animal studies

**Figure 1:**
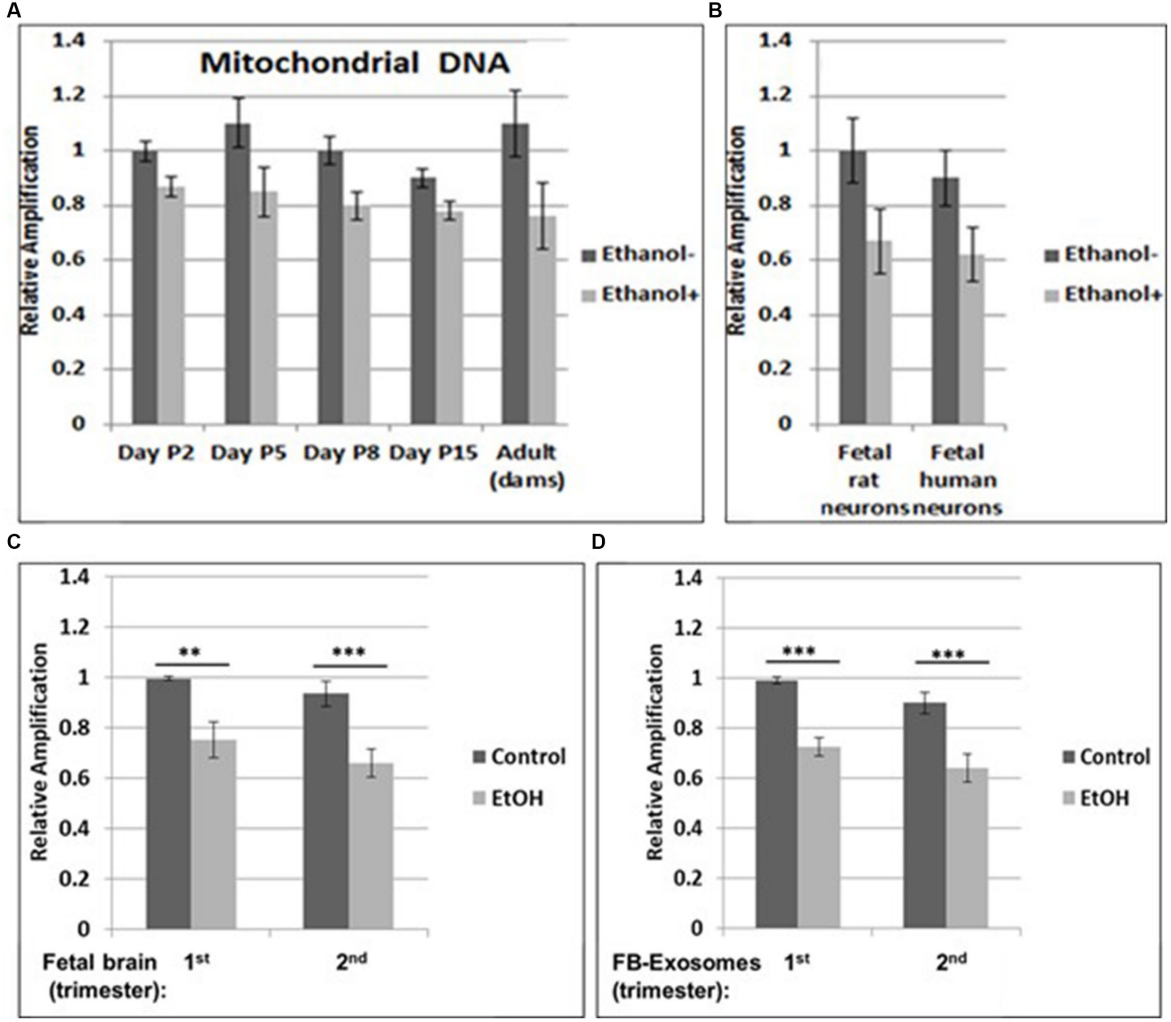
Increased mtDNA damage by EtOH. **(A)** Brain tissues from EtOH-fed rat pups (*n* = 4) were analyzed by qPCR with mitochondrial-specific primers, comparing long (16.5 kbp) and short (200) PCR products. **(B)** Fetal rat and human neuronal cells were incubated with EtOH (50 mM) or 48 h. **(C)** mtDNA damage in human fetal brains from two trimesters, and in fetal brain-derived exosomes **(D)**. mtDNA damage increased in human FB-Es from maternal blood from EtOH cases (*n* = 10/trimester). Relative amplification is shown for the mtDNA band intensities (treated/control [AD/AC] or damaged versus undamaged). The data are shown as the mean ± SD from three independent experiments. The decrease in relative amplification is shown by graphs. Assays were performed in triplicates and value of *p* was calculated using ANOVA and Student’s t-test.

Female time-pregnant Sprague–Dawley rats were maintained on a LieberDeCarli EtOH liquid Diet (Bio-Serv) (containing 6.7% EtOH v/v) or isocaloric Maltose Dextrin Control liquid diet (Bio-Serv) for 6 days until delivery. Dams and pups were maintained according to the Temple University Institutional Animal Care and Use Committee (IACUC)-approved protocol. Rats were given Liquid Rat Diet for Pregnant Dams, High Protein (BioServ) with addition of either enough 95% EtOH to reach a concentration of 6.7% concentration (for the EtOH group), or Maltose Dextrin (for the control group). Alcohol blood levels were not measured, but both groups consumed the same volume of food. The alcohol diet was stopped immediately after delivery. Rats were then monitored carefully through delivery and the first 15 postnatal days. Controls as well as ethanol-exposed pups were studied on their 2nd, 5th, 8th, and 15th postnatal day. Pups were weighed, measured for crown-to-rump length (using a soft measuring tape, forceps, and a ruler), analyzed for ocular and mtDNA abnormalities. Four rat pups (2 male and 2 female) were used for each timepoint (Figure 1). Hemi-brains were dissected for DNA studies. Eye globe diameter (lateral), eye length (anterior–posterior), and pupil diameter and shape were noted for both the right and left eyes of all fetuses and informative comparisons were made.

### 2.3. Cell culture

Human primary cortical neurons were prepared in our laboratory by Darbinyan et al. (2013a,b), Darbinian et al. (2014), De Simone et al. (2016), and Darbinian et al. (2021c). In brief, 16-weeks fetal brain (approx 13 g) was collected under an approved Temple University IRB protocol and was treated with Tryple Express enzyme (Invitrogen, CA), DNase I (10 U/mL; Sigma, St. Louis, MO) and maintained at 37°C.

#### 2.3.1. Cell treatment

Neuronal cells were incubated with 50 mM EtOH (Darbinian et al., 2021b), Insulin-like growth factor 1 (IGF-1, 50 ng/mL) or tumor necrosis factor α (TNFα, 50 ng/mL) for a total of 48 h.

### 2.4. RNA preparation and real-time qRT-PCR

Total RNA was isolated using the RNeasy kit (Qiagen, Valencia, CA) with on-column DNA digestion (Darbinian-Sarkissian et al., 2006). The RT-PCR reaction was performed with 1 μg total RNA, using One-Step FAST RT-PCR SYBR Green PCR Master Mix (Qiagen). StepOnePlus Real-Time PCR system was used (Applied Biosystems). PCR conditions were performed according (Darbinyan et al., 2013a, 2020). The amplified DNA was analyzed by gel electrophoresis using 2.0% agarose gel.

#### 2.4.1. cDNA synthesis

One μg of RNA and reverse transcriptase (Roche Molecular Biochemicals, Indianapolis, IN, United States) were used.

*RT-PCR.* The SuperScript III One-Step RT-PCR System with Platinum *Taq* (Invitrogen, Carlsbad, CA, United States) was used. One microgram of total RNA and primers specific to *ogg1* gene were used to amplify OGG1 (Darbinian et al., 2020).

### 2.5. Analysis of mtDNA damage by qPCR

qPCR for nuclear and mitochondrial DNA integrity was carried out with GeneAmp XL-PCR kit (Applied Biosystems, Darbinian et al., 2020).

### 2.6. Estimation of DNA damage

Quantification of PCR products and calculation of lesion frequency were done by using PicoGreen (Ahn, 1996; Kovalenko and Santos, 2009; Hunter et al., 2010; Gureev et al., 2017; Darbinian et al., 2020). DNA damage is indicated by lower signal for DNA (lower amplification).

### 2.7. DNA isolation and qPCR

Genomic DNA was purified using the QIAamp DNA isolation kit (Qiagen, Chatsworth, CA) to perform long PCR (Meyer, 2010; Furda et al., 2014; Darbinian et al., 2020). qPCRs was done in a GeneAmp PCR System 2,400 using the GeneAmp XL PCR kit. Fifteen ng of genomic DNA was used to perform long qPCR (see all details in Darbinian et al., 2020).

### 2.8. Primers (IDT Inc.)

See details in Darbinian et al., 2020: β-actin: S: 5’-CTACAATGAGCTGCG TGTGGC-3′,

AS: 5’-CAGGTCCAGACGCAGGATGGC-3′,

Primer nucleotide sequences for the 17.7-kb β-globin gene (GenBank: J0017 9J00179 ),

5′-TTGAGACGCATGAGACGTGCAG-3′, and 5′-GCACTGGCTTAGGAGTTGGACT-3′; and for the 16.2-kb fragment of the mitochondrial genome,

5′-TGAGGCCAAATATCATTCTGAGGGGC-3′ and.

5′-TTTCATCATGCGGAGATGTTGGATGG-3′ (RH1066). *mtDNA quantification.* Fluorescence of each product was detected at a wavelength of 530 nm. Relative concentrations of mtDNA were calculated for each mitochondrial transcript as the ratio of its average signal in triplicate assays to that of the housekeeping gene globin (Hunter et al., 2010; Darbinian et al., 2020). The quantitative loss (damage) or gain (repair) in fluorescence was detected during the PicoGreen analysis of control and treated QPCR products. DNA damage was quantified by comparing the relative amplification of long fragments (approximately 16 kb) of DNA from EtOH exposed or treated samples to controls, then by normalizing this to the amplification of short (200 bp) DNA fragments. If A_t_ represents the amplification of EtOH-treated samples and A_o_ is the amplification of untreated controls, then the relative change in mtDNA = A_t_/A_o_.

### 2.9. Droplet digital PCR (ddPCR)

For absolute quantitation of mRNA copies, ddPCR was performed using the QX200 ddPCR system. Fifty ng of human fetal total RNA were used with the 1st Strand cDNA Synthesis Kit (Qiagen, Valencia, CA, United States). All procedures were performed according to Darbinian et al. (2021a, 2023). The ddPCR data were exported to Microsoft EXCEL for further statistical analysis.

### 2.10. Isolation of Fetal brain-derived exosomes (FB-Es) from maternal plasma, and ELISA quantification of Exosomal proteins

Human FB-Es were isolated as described previously (Goetzl et al., 2016, 2019a; Darbinian et al., 2023). Two hundred and fifty μL of maternal plasma were used to precipitate with antihuman contactin-2/TAG1 antibody (clone 372,913, R&D Systems, Inc., Minneapolis, MN USA). Because our previous studies revealed that EtOH reduced the number of FB-Es, all exosomal assays were normalized against the exosomal marker CD81.

### 2.11. Superoxide dismutase activity assay

SOD activity was measured in primary neurons, incubated with EtOH for 48 h (Darbinian et al., 2020). Cells were analyzed by Superoxide Dismutase (SOD) assay by comparing SOD activity using the OxiSelect SOD Activity Assay kit. The activity of SOD is determined as the inhibition of chromogen reduction. In the presence of SOD, superoxide anion concentration is reduced, yielding less colorimetric signal. SOD activity is shown in % of control.

### 2.12. ATP assay

ATP level was measured using ATP Assay Kit (Darbinian et al., 2020). Human fetal neurons were incubated with EtOH or IGF-1. ATP was assayed using the ATP Determination Kit (Molecular Probes, Eugene, OR). Bioluminescence was measured using a Luminometer (Femtomaster FB 12 luminometer, Zylux).

### 2.13. Quantification of brain cell injury: caspase-GLO 3/7 activity assay

Apoptosis was assessed for activation of Caspase-3, using the Caspase-Glo™ 3/7 assay kit (Promega, Madison, WI, USA), according to the manufacturer’s instructions. Approximately 1,000 of rat pup’s brain cells, were analyzed in a final volume of 100 microliters culture medium per well. One hundred microliters of Glo reagent were added to the culture medium (1,1), then after cell lysis induction, the luminescence was recorded (RLU/s) on a Luminometer (Zylux Corporation). Data were analyzed using Excel software.

### 2.14. Microscopy

Human neurons were seeded in poly-L-Lysine coated glass slide chambers, and after 48 h incubation with 50 mM of EtOH, cells were analyzed by microscopy (Darbinian et al., 2021a). Phase-contrast images were captured using IPLAB software. Original magnification for phase images was 200x.

### 2.15. Statistical analysis

Statistical analysis was described previously (Darbinian et al., 2021a, 2023). In brief, analysis was performed using SPSS (IBM Corp., released 2017. IBM SPSS Statistics for Windows, Version 25.0. Armonk, NY). All data are represented as the mean ± standard error for all performed repetitions. Means were analyzed by a one-way ANOVA, with Bonferroni correction. Statistical significance was defined as *p* < 0.05. Sample numbers are indicated in the figure legends. More details are in Darbinian et al. (2021a, 2023).

### 2.16. Ethics statement

*Human Subjects.* Consenting mothers were enrolled between 11 - 21 weeks gestation, under a protocol approved by our Institutional Review Board (IRB). This protocol involved no invasive procedures other than routine care. Maternal EtOH exposure was determined with a face-to-face questionnaire that also included questions regarding many types of drugs/medications used (Goetzl et al., 2016, 2019a,b, Darbinian et al., 2021a, 2023). The questionnaire was adapted from that designed to identify and quantify maternal EtOH exposure in the NIH/NIAAA Prenatal Alcohol and SIDS and Stillbirth (PASS) study (Dukes et al., 2014).

All procedures for collection and processing of human brain tissues and blood were performed according to NIH Guidelines by a trained Study Coordinator. All investigators completed Citi Program - Human Subject training, Blood-Borne Pathogens Training, and Biohazard Waste Safety Training annually (see details in Darbinian et al., 2023).

Written informed consent has been obtained from the parents of patient(s) for studies, and deidentified samples were used for this publication. Informed Consent forms were maintained by the Study Coordinator. The de-identified log sheets contain an assigned accession number, and the age, sex, ethnicity, and race of the patient. Except for an assigned accession number, no identification was kept on the blood samples.

*Vertebrate Animals.* Experiments with rats were designed with the primary goal of minimizing the numbers of animals used. Toward this end, we have considered the minimal number of animals necessary for achieving statistical significance in all experiments (n = 4 for all time points). Animals were purchased and maintained in the medical school’s animal facility in accordance with all federal and state regulations on the humane care and use of animals in research, including the provisions of the Animal Welfare Act and the Public Health Service Policy on Humane Care and use of animals in research (PHS Policy on Humane Care and Use of Laboratory Animals, 2015, https://olaw.nih.gov/policies-laws/phs-policy.htm).

For tissue harvesting for RNA and protein, and behavioral studies, we utilized male and female rats which were monitored by animal facility personnel for 2 days prior to exposure to EtOH diet and then pups were sacrificed at 2-, 5-, 8- and 15-days postnatally.

Adult rats were euthanized by inhalation of CO2 without other manipulation, according to the recommendations of the Panel on Euthanasia of the American Veterinary Medical Association. Female pregnant rats were maintained on an EtOH-containing (6.7% v/v) liquid diet for 6 days until delivery, based on a Temple University IACUC-approved protocol.

## 3. Results

### 3.1. Increased mtDNA damage by prenatal EtOH exposure

Brain tissues from EtOH-fed rat pups were studied by qPCR with mitochondrial-specific primers, for relative mtDNA levels, comparing PCR products between EtOH-exposed groups and unexposed controls at four postnatal days. Increased mtDNA damage (lower relative amplification) was found in all EtOH groups (Figure 1A). A similar pattern of decreased mtDNA amplification was found in fetal rat and human cortical neurons (Figure 1B), and in human fetal brains (Figure 1C). Data from human fetal brains from 1st and 2nd trimesters (Table 1) were compared to results in fetal brain-derived exosomes isolated from matching maternal blood (Figure 1D). Relative mtDNA levels were lowest in EtOH groups.

Our previous data in primary OL cultures and fetal neural exosomes from EtOH-exposed maternal blood, demonstrate an association between EtOH injury and neuronal/OL markers. Molecular markers in FB-Es isolated during pregnancy can predict which at-risk fetus will develop FASD. Therefore, we isolated FB-Es from plasma to study the impact of EtOH exposure on mtDNA damage in the fetal brain, by measuring exosomal mtDNA damage, and a pattern of reduced mtDNA in FB-Es from EtOH-exposed plasma compared to control FB-Es. Interestingly, EtOH cases from both 1st and 2nd trimesters had more damaged mtDNA compared to non-exposed controls (Figures 1C,D). The similarity in the degree of EtOH-associated reduction in mtDNA levels between fetal brain and FB-Es shown in these graphs suggests that FB-Es from maternal blood might be useful as a surrogate marker for mtDNA in fetal brain.

### 3.2. Reduced cell viability and Sod activity By EtOH exposure

**Figure 2:**
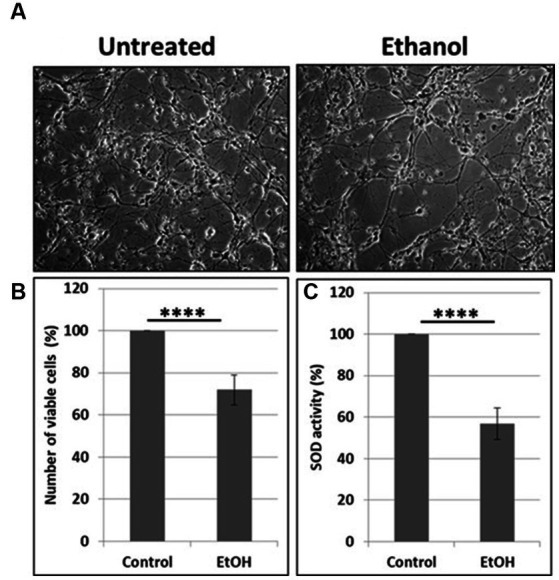
Reduction in cell viability and SOD activity. **(A)** Representative human neuronal cell images, incubated with EtOH (50 mM). **(B)** Cell viability assay in human neuronal cells. **(C)** SOD activity in neuronal cells presented as % control in human neuronal cells following exposure to EtOH (50 mM), with an OxiSelect SOD Activity Assay kit.

Neuronal cells with increased mtDNA damage by EtOH exposure (Figure 1B) were also studied for neuronal cell viability and SOD activity assays. EtOH treatment strongly affected cell survival, as only 72% of neuronal cells survived after 48 h of incubation with 50 mM EtOH (Figures 2A,B), probably in part, due to a reduced SOD activity (59%) in EtOH-treated cells (Figure 2C).

### 3.3. The neuroprotective growth factor, IGF-1, rescues mtDNA from damage caused By EtOH In neuronal cells

**Figure 3:**
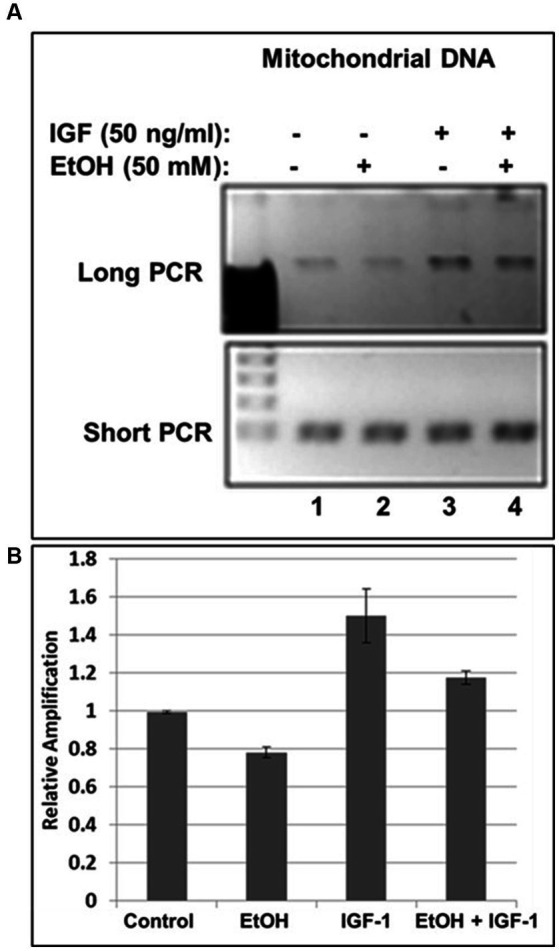
IGF-1 rescues mtDNA from the damage caused by EtOH. **(A)** Agarose gel images for long (16.2 kbp) or short (0.2 kbp) PCR products. Human neuronal cells were incubated with IGF-1 and EtOH. Total genomic DNA was isolated and long mtDNA qPCR was performed. **(B)** Relative amplification for mtDNA products.

Previously, we demonstrated neuroprotective role of IGF-1 in neuronal cells (Wang et al., 2014) toxic effects of EtOH on neuroprotective IGF-1 in fetal brain during development (Darbinian et al., 2021a), and suggested that downregulation of IGF-1 by EtOH exposure could be a cause of neuronal loss. Here, by exogenously adding IGF-1 to human neuronal cells, we studied the role of IGF-1 in rescuing mtDNA damage from EtOH. Incubation of cells with IGF-1 reverses EtOH-mediated mtDNA damage (Figure 3A). Interestingly, IGF-1 improved the quality of mtDNA when added to cells as shown in Figures 3A,B by comparing bar 3 to bar 1 (1.5-fold increase in mtDNA amplification).

### 3.4. Etoh downregulates mtDNA repair protein, OGG1, and IGF-1 reverses toxic effects of EtOH On OGG1 expression, and on mitochondrial energy metabolism In human neuronal cells

**Figure 4:**
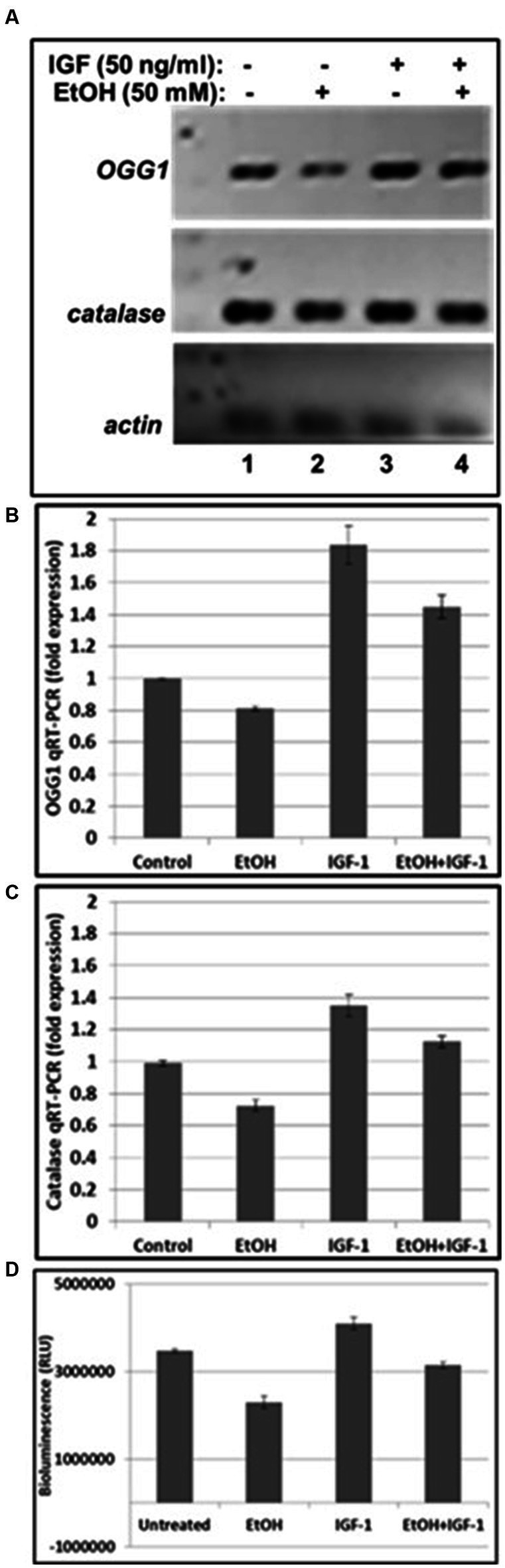
IGF-1 reverses toxic effects of EtOH on mtDNA repair protein in human cortical neurons. **(A)** qRT-PCR was performed using primers for mtDNA repair enzyme OGG1 and catalase. OGG1 is involved in the mtDNA damage repair mechanisms. Human neuronal cells were incubated either with EtOH, or IGF-1 alone or in combination, for 48 h. **(B)** Quantification of OGG1 qRT-PCR product. **(C)** Quantification of catalase qRT-PCR product. Results are presented in arbitrary units and shown as folds. **(D)** ATP activity. ATP assay was performed in neurons, incubated with EtOH, to demonstrate changes in ATPase activity.

Next, we assayed mtDNA damage repair protein, OGG1 mRNA expression, and studied whether neuroprotective IGF-1 can rescue mtDNA repair protein, and mitochondrial energy metabolism markers (catalase and ATPase activity) from EtOH caused toxic effects. As shown in Figures 4A,B, EtOH strongly downregulated OGG1 mRNA expression (compare bar 2 with bar 1), while IGF-1 reversed the toxic effects of EtOH on mtDNA repair protein (compare bar 4 with bar 2). Similar effects were seen for markers of mitochondrial energy metabolism, catalase (Figure 4C), and ATPase activity (Figure 4D).

### 3.5. Downregulation of the OGG1 mRNA expression by EtOH In fetal brain-derived exosomes

**Figure 5:**
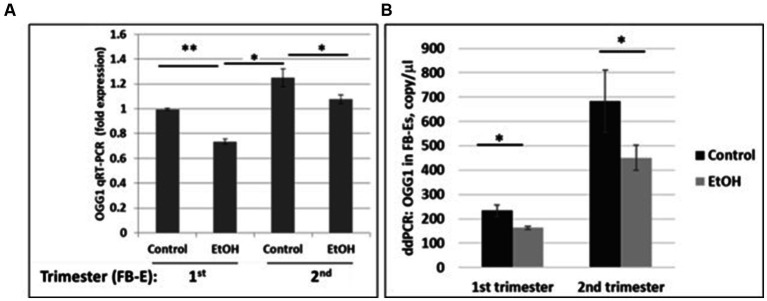
Downregulation of the OGG1 mRNA expression by EtOH in human fetal brain-derived exosomes (FB-Es). **(A)** Real-Time PCR for OGG1 mRNA in FB-Es. Downregulation was statistically significant (*p* < 0.05). Values are shown in fold expression (normalized to housekeeping *actin* gene). **(B)** ddPCR of OGG1 mRNA expression. Plasma from 20 patients with or without EtOH, were studied by ddPCR for OGG1 mRNA. Downregulation was greatest in the cases with EtOH exposure during 2nd trimester (graphs show means from triplicate assays +/− SD). Downregulation was statistically significant (*p* < 0.05). For absolute quantitation of OGG1 by ddPCR, values are shown in copies/μL.

We developed non-invasive methods to study effects of prenatal EtOH exposure on fetal brain development by isolating fetal brain-derived exosomes from maternal blood. Downregulation of the OGG1 mRNA expression in EtOH-exposed fetal brain-derived exosomes was assayed by RealTime PCR (Figure 5A). Downregulation was greatest in the cases with EtOH exposure. The downregulation of OGG1 mRNA by EtOH was studied by ddPCR for both trimesters (Figure 5B). A 44% downregulation of OGG1 mRNA expression was found in the 2nd trimester cases (451 copies/μL in EtOH FB-Es vs. 682 copies/μL in control exosomes).

### 3.6. IGF-1 rescues mtDNA from damage caused by both EtOH and neurotoxic TNFα in neuronal cells

**Figure 6:**
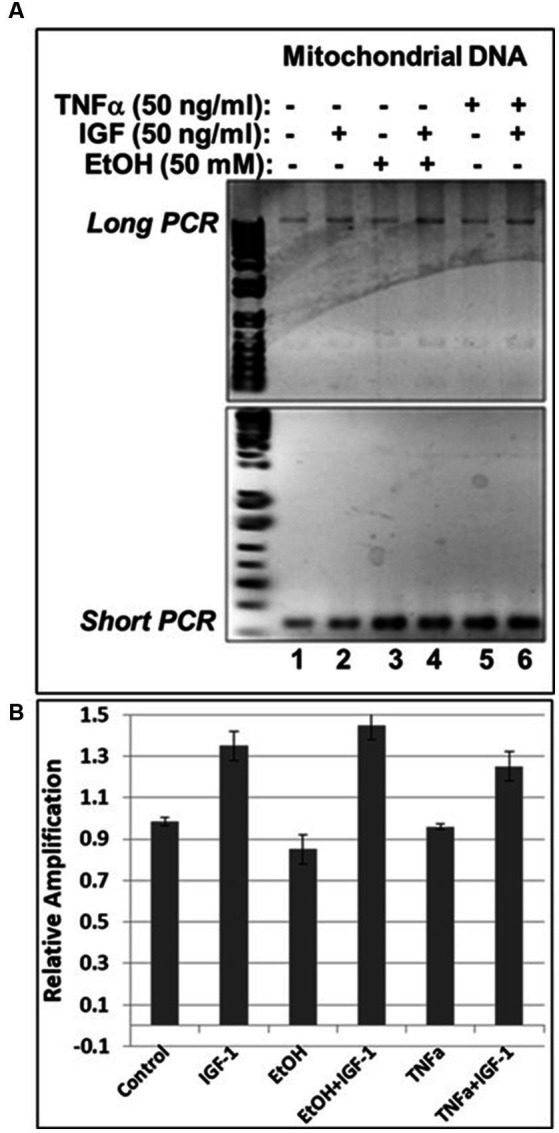
IGF-1 reverses mtDNA damage caused by EtOH or neurotoxic TNFα toxicity. **(A)** Agarose gel images for mtDNA amplification products. Human neuronal cells were incubated with EtOH, IGF-1 and TNFα alone or in combination and incubated for 48 h. **(B)** mtDNA damage was measured and presented as a relative amplification (graphs show means from triplicate assays +/− SD).

In our previous studies we demonstrated that prenatal EtOH exposure not only inhibits neuroprotective IGF-1, but also upregulates neurotoxic TNFα in fetal brains (Darbinian et al., 2021a). Here, we demonstrate the protective role of IGF-1 in the direct effect of EtOH on mtDNA damage, and on the EtOH-associated indirect effect, via TNFα, as revealed by qPCR assays in human cortical neuronal cells (Figure 6). IGF-1 efficiently reversed EtOH-caused mtDNA damage (compare lane 4 with lane 3 in Figure 6A, or bar 4 with bar 3 in Figure 6B). Similar protective effects were seen for IGF-1 in TNFα-treated cells (compare lane 6 with lane 5 in Figure 6A, or bar 6 with bar 5 in Figure 6B).

### 3.7. IGF-1 protects human neuronal cells from loss caused by EtOH and TNFα

To investigate the impact of IGF-1 on cell survival, human cortical neuronal cells were incubated with IGF-1, TNFα or EtOH (50 mM) alone or in combinations, for 48 h, and a cell viability assay was performed (Figure 6A). Reduction in number of neurons was seen in cases with EtOH treatment (68.3%), and TNFα incubation (72.3%), while IGF-1 reversed the toxic effects on cell survival from both EtOH- (89%) and TNFα- (92%) treated cells. Representative cell images of TNFα- and EtOH-treated cells used for the cell survival assay are also presented (Figure 6B).

### 3.8. Downregulation of IGF-1 mRNA and upregulation of TNFα mRNA by EtOH In FB-Es from patients with EtOH exposure

**Figure 7:**
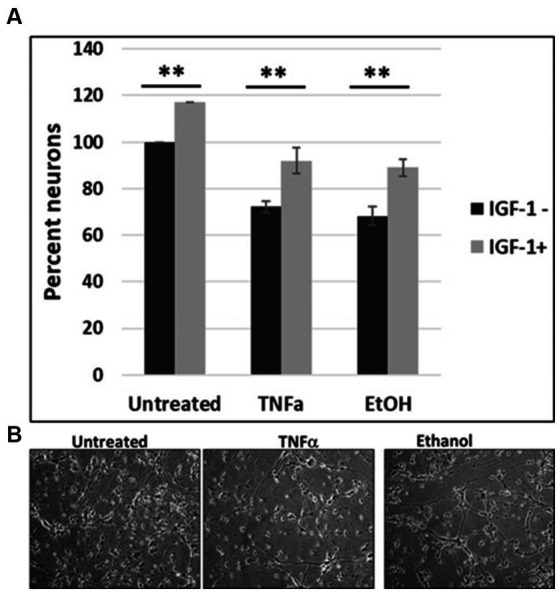
IGF-1 reverses neuronal loss caused by EtOH or TNFα toxicity. **(A)** Human cortical neurons were incubated with IGF-1, TNFα or EtOH (50 mM) alone or in combinations, for 48 h, and a cell survival assay was performed. Reduction in the number of neurons was assayed by a cell viability assay. Means and SD of the % of cells. Untreated = 100%. **(B)** Representative cell images.

**Figure 8:**
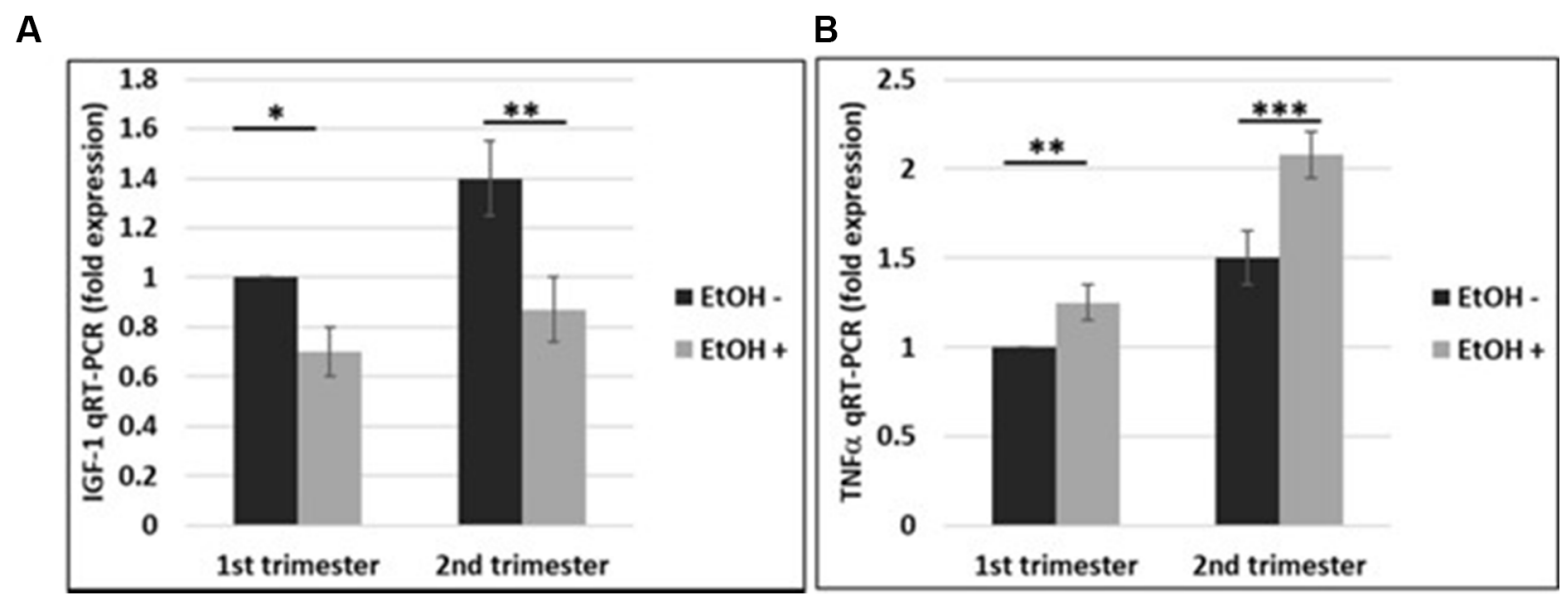
EtOH downregulates IGF-1 and upregulates TNFα in human fetal brain-derived exosomes. **(A)** Downregulation of the IGF-1 mRNA in FB-Es from EtOH-exposed cases. FB-Es from plasma were measured for IGF-1 mRNA by qRT-PCR, using specific primers for IGF-1. Downregulation was greatest in the cases with EtOH exposure (graphs show means from triplicate assays +/− SD). Downregulation was statistically significant (*p* < 0.05). **(B)** qRT-PCR was performed for TNFα in the same FB-Es. Plasma from 20 patients (*n* = 10 from 1st trimester, and *n* = 10 from 2nd trimester) with or without EtOH, were used for isolating FB-Es, and studied by Real-Time PCR. Upregulation was greatest in the cases with EtOH exposure (graphs show means from triplicate assays +/− SD). Upregulation was statistically significant (*p* < 0.05).

The effects of EtOH exposure on IGF-1 and TNFα expression seen in fetal brain tissues (Darbinian et al., 2021a) and human cortical neurons (Figure 7), were reproduced in FB-Es. qRT-PCR was performed for both markers during the 1st and 2nd trimesters. There was a significant downregulation of IGF-1 by EtOH (Figure 8A). TNFα was upregulated in FB-Es from the EtOH group in both trimesters (1.25-fold and 2.08-fold respectively).

Thus, we were able to reproduce data previously obtained in fetal brains (Darbinian et al., 2021a) in human cortical neurons (Figure 7), and in fetal brain-derived exosomes from maternal blood, indicating availability of a novel well-developed non-invasive tool to study events in fetal brain by using maternal plasma during pregnancy.

### 3.9. Correlations between rat pup Eye size and mtDNA damage. Prenatal alcohol exposure is associated with increased caspase-3 activity and morphological alterations in the rat

**Figure 9:**
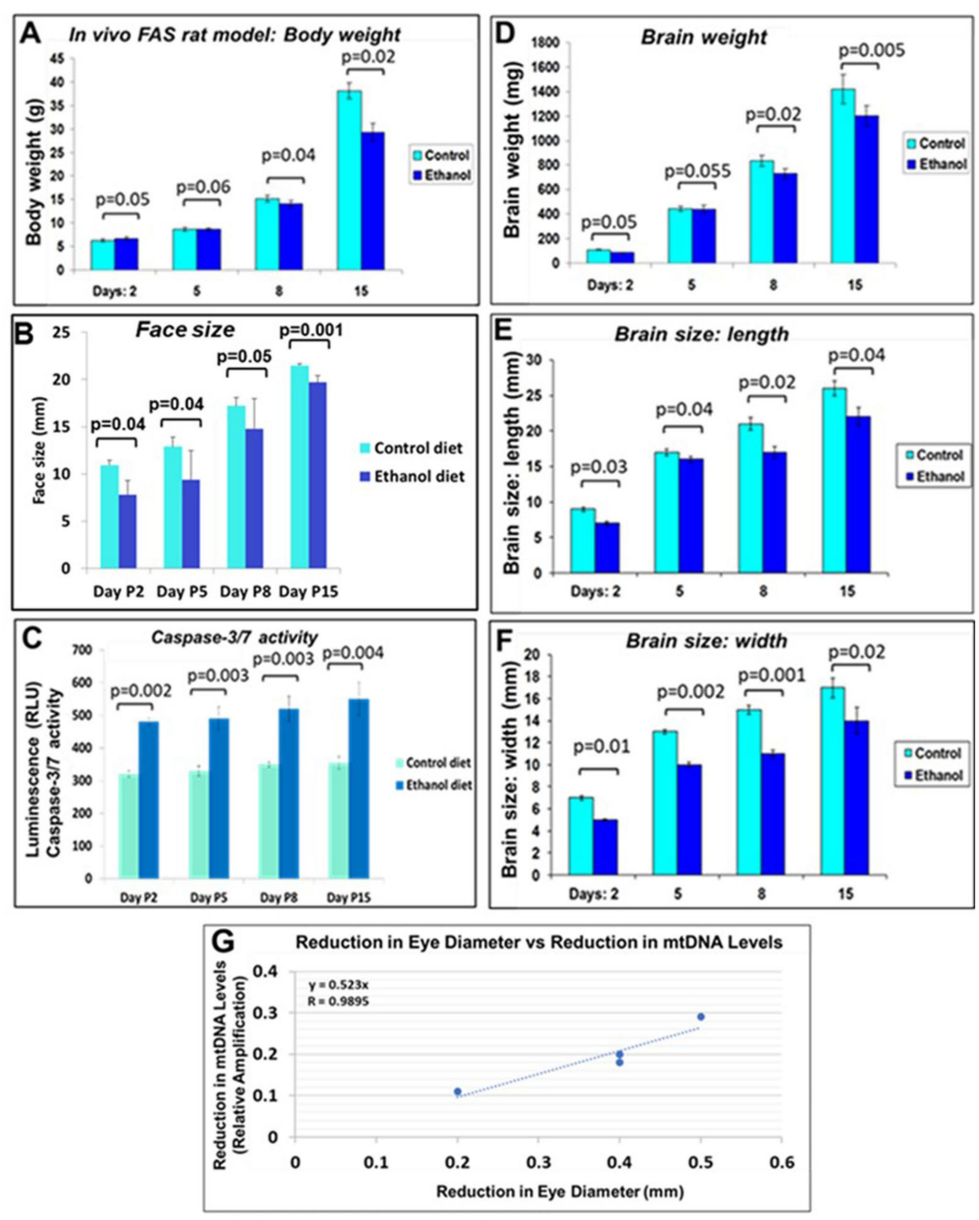
Correlations between rat pup eye size and mtDNA damage. Effect of prenatal EtOH exposure on morphological phenotype and caspase-3 activity in rat pups from age P2-P15. Rat dams were fed a liquid diet with or without 6.7% EtOH, and the effect on rat pup developmental parameters measured from postnatal days 2–15. **(A)** Negative effect of prenatal EtOH intake by dams on the body weight of their pups. **(B)** Effect of EtOH exposure and gestational age on pup face size increases during development. Measurements were performed using a soft measuring tape, forceps, and a ruler (mm). **(C)** EtOH diet increased brain caspase-3/7 activity at all developmental stages. Tissue lysates prepared from brains of untreated pups, and pups treated with EtOH, were analyzed for cleaved (active) caspase-3, assessed with the substrate DEVD amino luciferin in the Caspase-Glo™ 3/7 assay kit (Promega, Madison, WI, United States). Luminescence was recorded as RLU/s. The histogram shows caspase 3/7 activity, the error bars show the standard deviation from three independent readings. **(D)** Delayed reduction in brain weight in EtOH-exposed pups from P2-P15. **(E)** EtOH exposure reduced brain length in pups. **(F)** EtOH reduced brain width. **(G)** Correlations between pups’ eye sizes and mtDNA levels in brain. Eye diameters were measured in histological sections. mtDNA levels were measured by qPCR. At each time point from postnatal days 2, 5, 8, and 15, eye tissues from four EtOH-exposed pups were paired with age-matched controls. Correlation between reduction in eye size (difference between EtOH-exposed pups and its paired control) and reduction in mtDNA levels presented as a scatter plot. Calculations in **(G)** are based on Spearman’s correlation on exact Two-Tailed Probabilities critical *p* values for *N* > 2 < =18, estimated using the Edgeworth approximation (Ramsey, 1989; Fowler et al., 2009).

We investigated effects of alcohol on rat pup eye formation in the *in vivo* rat model of FAS. There was a negative correlation between prenatal EtOH intake by dams (6.7% liquid diet) and several parameters of the development of pups (Figure 9). The body weight of pups prenatally exposed to EtOH was lower than that of the control group at postnatal days 8 and 15 (P8 and P15; Figure 9A). At P2, the body weight of pups from the ethanol group was slightly higher than that of the control group. This difference was reversed at days P5 and P8. EtOH exposure also was associated with decreased weight and size (length and width) of the pups’ brains and decreased face size. These effects were most pronounced at later GAs. We also examined the effect of prenatal EtOH exposure on apoptotic activity in the brain tissue. Lysates prepared from brains of pups, either untreated or treated with EtOH, were analyzed for cleaved active caspase-3/7 using the GLO caspase-3/7 apoptotic assay. Luminescence was recorded as RLU/s. The Glo assay results depicting caspase 3/7 cleavage in cells after EtOH diet are shown in Figure 9C. The values represent the readings from the Luminometer. Compared to controls, there was an increase in active caspase-3 levels in the brains of the EtOH groups at all developmental stages.

### 3.10. Prenatal EtOH exposure delays body and eye development in rat pups

A negative correlation was found between prenatal ethanol uptake by dams (6.7% liquid diet) and anatomical parameters of their pups (Table 2). Comparative measurements of the body length and eye size of EtOH diet and control diet rat pups, and other morphological changes, confirmed the toxic effects of EtOH on eye development. The body length of pups from the ethanol group was slightly less than that of the control group at day P2. This difference also was found on day P15. Thus, a consistent pattern of reduced eye size was observed with EtOH exposure in the rat pups.

**Table 2:** Negative correlation between prenatal EtOH intake by rat dams (6.7% liquid diet) and the development of rat pups.

	Body length, cm (tail not included)	Eye diameter, mm
Control P2, *n* = 4 (±SD)	3.5 ± 0.2	3.5 ± 0.1
FAS P2, *n* = 4 (±SD)	3.0 ± 0.1	3.1 ± 0.2
Control P15, *n* = 4 (±SD)	5.5 ± 0.2	4.0 ± 0.3
FAS P15, *n* = 4 (±SD)	5.0 ± 0.3	3.8 ± 0.2
Male Fetal Sex (%)	50	50

Comparative measurements of the body length and eye size of the FAS (EtOH diet) and Control (control diet) rat pups. The n for each group represents the number of rat pups. For each pup, both eyes were measured, and the mean diameter was used for the calculations in the table.

Negative correlation was between prenatal EtOH intake by dams and the body weight of their pups (Figure 9A), pup face size (Figure 9B). Measurements (mm) were performed using a soft measuring tape, forceps and a ruler. EtOH diet increased brain caspase-3/7 activity at all developmental stages (Figure 9C). Tissue lysates prepared from brains of untreated pups, and pups treated with EtOH, were analyzed for cleaved (active) caspase-3, assessed with the substrate DEVD-aminoluciferin in the Caspase-Glo™ 3/7 assay kit. Delayed reduction in brain weight in EtOH-exposed pups from P2-P15 was measured (Figure 9D). EtOH also reduced brain length in pups from the EtOH group (Figure 9E) and brain width (Figure 9F). Correlations between pups’ eye size and mtDNA damage in brain was significant (Figure 9G), while similar analysis of pups’ brain width vs. mtDNA damage failed to show statistically significant differences.

### 3.11. Correlations between human fetal Eye size and fetal brain-derived exosomal mtDNA damage

**Figure 10:**
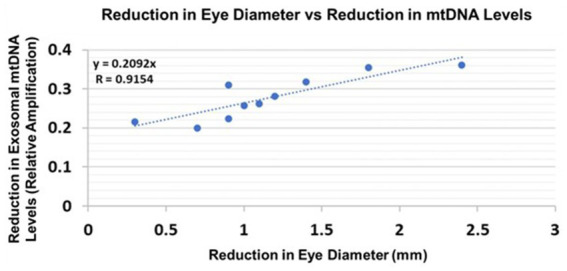
Correlations between human fetal eye size and fetal brain-derived exosomal mtDNA levels. Eye diameters were measured in histological sections. mtDNA levels were measured by qPCR (for relative amplification). Ten EtOH-exposed fetal eye tissues each were paired with an age- and sex-matched control, and with their matching maternal blood samples from 1st and 2nd trimester pregnancies (*n* = 20 total). Assays were performed in triplicate on contents of FB-Es isolated from the maternal blood. Correlation between reduction in eye size (difference between EtOHexposed fetus and its paired control) and reduction in mtDNA levels presented as a scatter plot. Calculations are based on Spearman’s correlation on exact Two-Tailed Probabilities critical p values for *N* > 2 < =18 (Ramsey, 1989; Fowler et al., 2009).

Eye diameters were measured in histological sections (Darbinian et al., 2023). mtDNA levels were measured by qPCR (for relative amplification). Correlation between change in eye size (difference between EtOH-exposed fetus and its paired control) and reductions in exosomal mtDNA amplification for mtDNA damage levels was significant (Figure 10).

## 4. Discussion

Although FASD is diagnosed in only a small number of children exposed prenatally to EtOH, the frequency with which FASD is diagnosed has been increasing. Unfortunately, FASD remains underdiagnosed. The present study demonstrates that mtDNA and mitochondrial energy metabolism markers in FB-E can suggest the presence of FASD very early during fetal development. These exosomes can be isolated noninvasively from maternal blood samples.

The contents of FB-E isolated from the blood of pregnant women who consumed EtOH revealed an increase in mtDNA damage. Thus, future clinical studies could be aimed at using FB-Es from maternal blood to detect fetal mtDNA damage early in pregnancies and whether this can predict FASD in postnatal at-risk children.

We previously established primary cultures of neurons and OLs (Amini et al., 2009; Darbinyan et al., 2013a,b, 2021; Darbinian et al., 2021a,b), and showed that in these cultures, and in FB-E from EtOH-exposed maternal blood, there was an association between EtOH exposure and several brain cell markers (Goetzl et al., 2016, 2019a; Darbinian et al., 2021a, 2023). These studies have now been extended to demonstrate that FB-Es from EtOH-exposed fetuses can carry additional markers for abnormalities of mtDNA and mitochondrial function pathways (e.g., low levels of mtDNA, OGG1 mRNA, and low levels of IGF-1), and thus may be a source of additional molecular markers for FASD. As far as we know, these are the first EtOH-exposed cases that have been studied for mtDNA and mitochondrial markers, OGG1, and IGF-1 using FB-Es.

We showed the effects of EtOH exposure on mtDNA damage in primary cortical rat and human neurons, in fetal brain tissues, and in fetal brain-derived exosomes (FB-Es). Our FB-E noninvasive method can enable studies of mtDNA damage affected by EtOH and other neurotoxic agents with further translational impact. We determined whether molecules in these fetal-derived exosomes can predict which at-risk fetuses will be born with FASD, the most common cause of developmental intellectual disability in the US, affecting quality of life by causing early aging. Non-invasive *in utero* diagnosis could lead to earlier detection and better research in therapies to prevent or ameliorate FASD.

Previously, we and others identified that IGF-1 in EtOH-exposed human subjects and *in vitro* studies regulate neuronal and OL injury and survival (Wang et al., 2014; Darbinian et al., 2021a; Darbinian and Selzer, 2022). IGF-1 improved mitochondrial function and reduced mitochondrial generation of reactive oxygen species (Ribeiro et al., 2014). IGF-1 protected OLs from TNFα-induced injury (Ye and D'Ercole, 1999; Lagarde et al., 2007), while OL progenitors of IGF-1-deficient mutant mice did not accumulate, proliferate, or survive. Thus, IGF-1 signaling plays an essential role in remyelination (Mason et al., 2003). IGF-1 also has been tested for treatment of neurological diseases. Five controlled clinical studies have evaluated human recombinant IGF-1 (rhIGF-1) as a treatment of ALS and Rett syndrome. These findings have been strengthened and expanded in the present study, in which we demonstrated: (i) Levels of the generally neuroprotective peptide IGF-1 were reduced in EtOH-exposed FB-Es, while levels of the generally neurotoxic peptide TNFα were increased; (ii) mtDNA damage was increased in fetal brain and neuronal cultures exposed to either EtOH or TNFα; (iii) IGF-1 protected neurons from EtOH-associated apoptosis; (iv) IGF-1 protected mtDNA from the effects of EtOH or TNFα.

We do not yet know which of the biomarkers, including those studied in our previous work (Goetzl et al., 2016, 2019a; Darbinian et al., 2023) and in the present investigation, will turn out to be the most accurate and sensitive predictors of FASD in at-risk infants. However, the present findings suggest that damage to mtDNA might be a promising biomarker because its measurements in FB-Es revealed a significant correlation between the increase in mtDNA damage and magnitude of one of the anatomical phenotypic hallmarks of FASD, i.e., reduced eye size (Hoyme et al., 2016). This was true for both rat pups (Figure 9) and human fetuses (Figure 10). A similar correlation had been found previously between MBP levels and reduced eye diameter (Darbinian et al., 2023). Large scale prospective studies in pregnancies brought to term, could test the diagnostic value of FB-E cargos, including those relating to mtDNA and mitochondrial repair, in predicting the emergence of FASD postnatally.

### 4.1. Limitations

Quantification of EtOH consumption was based on the subjects’ information in face-to-face interviews, not on biochemical assays, e.g., blood alcohol levels or hair ethyl glucuronide (EtG) levels. This is because such tests remain positive only for a limited time after cessation of alcohol exposure (blood and urine EtG levels detect alcohol use only for the previous few days to weeks), although EtG can be detected in hair for up to 90 days (Berger et al., 2014). At best, these tests could verify recent alcohol use, but could not rule out early prenatal use, and certainly not quantify it, and it seems more likely that subjects might deny use than falsely report use of alcohol. A relative’s opinion also was not always available for confirmation of alcohol use, particularly given the personal nature of the decision to terminate the pregnancy. We used another questionnaire to exclude exposure to SSRIs, opioids, marijuana and other medications and drugs. In general, we were confident about most of the subjects’ information in this patient population, because in previous studies, regular use of other drugs was confirmed by biochemical assays.

We did not validate our animal model by performing behavioral studies. Initially, we focused our animal studies on molecular, not behavioral effects of EtOH exposure. Later we obtained grant support to perform human exosomal studies, which have led to the observations reported here. Future clinical studies are planned to determine whether the molecular markers suggested by the present and other studies from our laboratory can predict which at-risk infants will develop FASD.

## 5. Conclusion

The present findings on human and murine tissues suggest involvement of mtDNA damage in EtOH-exposed fetal brain. The results on FB-E and on neuronal cultures were similar, and thus have the potential to be extended to non-invasive diagnostic analyses of fetal development that could predict the emergence of FASD prenatally. As a corollary, the data on FB-E from the blood of 20 pregnant women who drank EtOH, and 20 non-drinking controls, showed that gene expression for the DNA repair protein OGG1 and for IGF-1 was lower in EtOH-exposed fetuses, while gene expression for TNFα was higher, and these results were reproduced in fetal cortical neuronal cultures. Neuronal viability *in vitro* was reduced by EtOH, as was SOD activity, ATPase activity and catalase mRNA expression. All these findings are consistent with the hypothesis that global mitochondrial pathways are significantly impaired in fetuses exposed to EtOH. The findings of reduced IGF-1 levels in EtOH-exposed fetuses suggest the need for follow-up studies to determine whether IGF-1 administration might help protect fetuses exposed to EtOH, as previously suggested for EtOH-induced neuronal and OL injury (Darbinian et al., 2021a, 2023). The knowledge derived could lead to use of FB-E cargo assays as diagnostic and prognostic tools for the development of therapies to prevent or treat FASD and early aging. That this might be so is suggested by the close correlation found between the increase in mtDNA damage in FB-Es and an anatomical hallmark of FASD, i.e., the reduction in eye diameters demonstrated in both rat and human fetuses.

## Data availability statement

The raw data supporting the conclusions of this article will be made available by the authors, without undue reservation.

## Ethics statement

The studies involving human participants were reviewed and approved by Temple University Institutional Review Board (IRB). The patients/participants provided their written informed consent to participate in this study. The animal study was reviewed and approved by Temple University Institutional Animal Care and Use Committee (IACUC)-approved protocol.

## Author contributions

ND: designing of the experiments, developing exosome studies, managing the project, supervising all experimental processes, writing of the first draft, participation in reviewing and editing the manuscript, and supporting the project financially. AD: conceptualization, idea, methodology, data analysis, writing, reviewing, and editing, and visualization. NM: flow cytometry analysis, supervising students, participated in exosome isolation, and editing of the manuscript. MK: discussion, editing, and carrying out data collection. GT: statistical support. SA: reviewing and editing the manuscript and supporting the project financially. LG: reviewing, and editing of the manuscript, developing exosome studies, and supporting the project financially. MS: designing of the study and interpretating the data, provided overall scientific expertise, participated in the writing of the first draft of the manuscript, reviewing and editing the final manuscript, and supporting the project financially. All authors have read and approved the last version of the manuscript.

## Funding

This work was supported by NIH grant to SA; NIH grant R01HD069238, and Gates Foundation grant OPP1119489 to LG, by NIH grants R01NS97846, R01NS097846-02S1 and R01NS092876 awarded to MS; Shriners research grant SHC-85400 awarded to MS; and United States Pennsylvania State Health Department grant Project 10: 420491–04400-02 to ND.

## Conflict of interest

The authors declare that the research was conducted in the absence of any commercial or financial relationships that could be construed as a potential conflict of interest.

## Publisher’s note

All claims expressed in this article are solely those of the authors and do not necessarily represent those of their affiliated organizations, or those of the publisher, the editors and the reviewers. Any product that may be evaluated in this article, or claim that may be made by its manufacturer, is not guaranteed or endorsed by the publisher.
